# KLaR: fusing knowledge graphs and language models for biomedical target discovery

**DOI:** 10.1093/bioinformatics/btag320

**Published:** 2026-07-07

**Authors:** Yinghui Jiang, Zixian Li, Yanchao Xu, Haotong Sun, Bocheng Xu, Xiangrong Liu

**Affiliations:** National Institute for Data Science in Health and Medicine, Xiamen University, Xiamen, Fujian 361102, China; Department of Computer Science and Technology, Xiamen University, Xiamen, Fujian 361005, China; Shenji Technology Co., Ltd, Hangzhou 310000, China; Shenji Technology Co., Ltd, Hangzhou 310000, China; Shenji Technology Co., Ltd, Hangzhou 310000, China; Shenji Technology Co., Ltd, Hangzhou 310000, China; Shenji Technology Co., Ltd, Hangzhou 310000, China; National Institute for Data Science in Health and Medicine, Xiamen University, Xiamen, Fujian 361102, China; Department of Computer Science and Technology, Xiamen University, Xiamen, Fujian 361005, China

## Abstract

**Motivation:**

Biomedical knowledge relevant to disease mechanisms and therapeutic discovery is distributed across structured knowledge graphs (KGs) and unstructured text. Although pretrained language models provide strong semantic embeddings, adapting them to biomedical link prediction typically requires domain-specific fine-tuning and may weaken explicit structural constraints. An open challenge is to enhance link prediction with principled knowledge–language fusion while keeping language encoders lightweight and controllable.

**Results:**

We propose KLaR, a knowledge–language representation framework for biomedical KG link prediction. Given a query entity, KLaR encodes its local *k*-hop neighborhood with a relational GNN and constructs mechanism-consistent textual contexts by template-based textualization of random-walk paths within the same subgraph. These contexts are embedded using a frozen sentence-embedding model (without external retrieval or task-specific fine-tuning), and structural and textual views are aligned and fused via gated integration. To model heterogeneous biomedical interactions, KLaR uses a sparse mixture-of-experts decoder for triple scoring. On PharmKG, HetioNet, and DTINet, KLaR yields consistent gains over structure-only and KG–language hybrid baselines under standard filtered evaluation. We additionally report text-only LLM reference results under a candidate-restricted protocol due to the infeasibility of full-entity ranking for prompted generation. Case studies suggest that KLaR can recover biologically plausible disease–gene and drug–target associations missing from the original graphs, supporting hypothesis generation without domain-specific language model fine-tuning.

**Availability and implementation:**

Code and processed data are available at: https://github.com/stardj/KLaR.

## 1 Introduction

Large-scale biomedical knowledge graphs (KGs) that integrate curated information about diseases, drugs, genes, and other entities have become indispensable resources for target discovery and drug repositioning (Himmelstein *et al.* 2017, Zheng *et al.* 2021). By explicitly encoding heterogeneous relations such as disease–gene and drug–target interactions, KGs provide a structured substrate for organizing and querying biomedical knowledge. At the same time, a substantial amount of complementary biomedical information is expressed in textual form, including scientific literature and clinical reports. Effectively integrating these heterogeneous sources of information into a unified representation remains a central challenge for biomedical knowledge modeling and reasoning.

A large body of work has explored knowledge graph embedding (KGE) and graph neural network (GNN) models for link prediction in biomedical KGs (Bordes *et al.* 2013, Trouillon and Bouchard 2017, Schlichtkrull *et al.* 2018, Sun *et al.* 2019, Vashishth *et al.* 2020). Classical KGE methods operate purely on observed graph structure, while more recent GNN-based approaches propagate messages along heterogeneous edges to learn context-aware representations. Although these models have achieved promising performance, they rely exclusively on structural evidence encoded in the KG and are unable to directly incorporate complementary semantic information available in textual form.

In parallel, domain-specific language models (LMs) such as BioBERT (Lee *et al.* 2020) and more recent large language models (LLMs) provide powerful semantic representations learned from large-scale biomedical corpora. These models implicitly encode rich information about disease mechanisms, molecular pathways, and therapeutic effects. However, effectively adapting LMs to biomedical knowledge graph reasoning often requires extensive domain-specific fine-tuning and lacks explicit structural constraints, making it challenging to integrate them into standard KG link prediction pipelines.

Recent work has therefore attempted to combine KGs with language models (Yao *et al.* 2019, Wang *et al.* 2021, Wang *et al.* 2022), e.g. by augmenting node representations with textual descriptions or refining KG embeddings using pretrained language models. While such KG–LM hybrid approaches demonstrate the benefits of incorporating textual information, most existing methods treat graph structure and text as loosely coupled signals, or rely on large, fine-tuned language models as the primary source of reasoning. As a result, it remains difficult to disentangle the contribution of information fusion itself from the effects of model scale and extensive fine-tuning.

In this work, we propose **KLaR**, a Knowledge–Language Representation framework for biomedical link prediction that performs principled information fusion between local graph structure and textualized neighborhood context in a lightweight manner. Given a target entity, KLaR first extracts its *k*-hop neighborhood and encodes this local subgraph using a relational attention-based GNN, producing a structure-centric embedding. In parallel, KLaR converts multiple random-walk paths within the same neighborhood into natural-language fragments via a template-based textualization module, and embeds the resulting context using a *frozen pretrained sentence-embedding model*. This design enables effective domain reasoning through information enhancement, without relying on task-specific language model fine-tuning. The structural and textual views are projected into a shared space, softly aligned, and fused through a gating mechanism. To model the heterogeneity of biomedical interactions, KLaR uses a sparse Mixture-of-Experts (MoE) decoder, allowing different experts to specialize in distinct relation patterns while maintaining inference efficiency.

Our contributions are summarized as follows:

- **Mechanism-consistent graph–text information fusion.** We introduce a principled framework that aligns GNN-based structural encodings and embedding-based textual representations at the level of local neighborhoods, rather than at the node or description level. This design grounds both modalities in the same subgraph context, enabling coherent information enhancement for biomedical link prediction.
- **Relation-aware sparse Mixture-of-Experts decoding.** We model heterogeneous biomedical relations using a sparse MoE decoder as a mechanism-specialized inductive bias, allowing different experts to capture distinct interaction patterns beyond what a single shared scoring function can represent.
- **Efficient biomedical reasoning without domain-specific language model fine-tuning.** Experiments on three heterogeneous biomedical KGs (PharmKG, HetioNet, and DTINet) show that KLaR achieves competitive and often superior performance compared to strong KGE, GNN, and KG–LM baselines, while relying only on a frozen 0.6B-parameter embedding model. Case studies further demonstrate that KLaR can recover biologically plausible disease–gene and drug–target associations absent from the original graphs, supporting hypothesis generation under a lightweight and controllable modeling paradigm.

## 2 Related work

### 2.1 Structure-based biomedical KG representation learning

Traditional knowledge graph embedding (KGE) methods, such as TransE (Bordes *et al.* 2013), RotatE (Sun *et al.* 2019), and ComplEx (Trouillon *et al.* 2016), learn latent representations solely from observed triples. To capture higher-order dependencies, graph neural networks (GNNs) have been widely adopted for relation-aware message passing over heterogeneous graphs. In the biomedical domain, models such as Bio GCN, MEKGNN (Wu *et al.* 2024), and DTD-GNN (Li *et al.* 2024b) aggregate neighborhood information to produce context-aware embeddings for tasks such as drug–target interaction and disease association prediction. While effective at modeling graph structure, these approaches rely primarily on KG-derived signals and do not explicitly incorporate complementary semantic information from text.

### 2.2 Language models and text-augmented KG completion

Biomedical language models, including BioBERT (Lee *et al.* 2020), BioGPT (Luo *et al.* 2022), and more recent large language models (Touvron *et al.* 2023), provide strong semantic representations learned from large-scale corpora. A parallel line of work therefore augments KG completion models with textual information, e.g. through entity descriptions or pretrained text encoders, as in KG-BERT (Yao *et al.* 2019), BioBLP (Daza *et al.* 2023), and FuseLinker (Xiao *et al.* 2024). These methods demonstrate the value of textual signals, but most either couple graph and text only loosely, operate mainly at the node-description level, or rely on computationally expensive fine-tuning of large language models.

### 2.3 Position of KLaR

KLaR differs from prior work in two ways. First, it performs *mechanism-consistent* fusion by deriving both structural and textual views from the same local neighborhood subgraph, rather than combining a graph encoder with external or weakly grounded text. Second, it uses a sparse relation-aware Mixture-of-Experts decoder as an inductive bias for heterogeneous biomedical relations, while keeping the text encoder frozen and shifting task-specific reasoning to the fusion and decoding modules. This design provides a compact alternative to full autoregressive language-model inference for standard biomedical KG link prediction.

## 3 Materials and methods

We propose **KLaR**, a framework that integrates local topological structure with textualized biomedical context via a mechanism-specialized architecture. Formally, we address the problem of link prediction in a biomedical knowledge graph G=(V,R,E), where V is the set of biological entities, R is the set of relation types, and E⊆V×R×V is the set of observed triples. Our goal is to learn a scoring function f:V×R×V→R that ranks unobserved true triples higher than false ones. Importantly, all textual information used in KLaR is strictly derived from local subgraph context rather than external biomedical corpora.

Distinct from approaches that rely on generative language models for inference-time reasoning, KLaR decouples semantic encoding from relational scoring. It utilizes a frozen pretrained encoder as a feature extractor, shifting task-specific reasoning to a compact, mathematically grounded fusion encoder and a Mixture-of-Experts (MoE) decoder.

### 3.1 Model architecture overview

**Figure 1:**
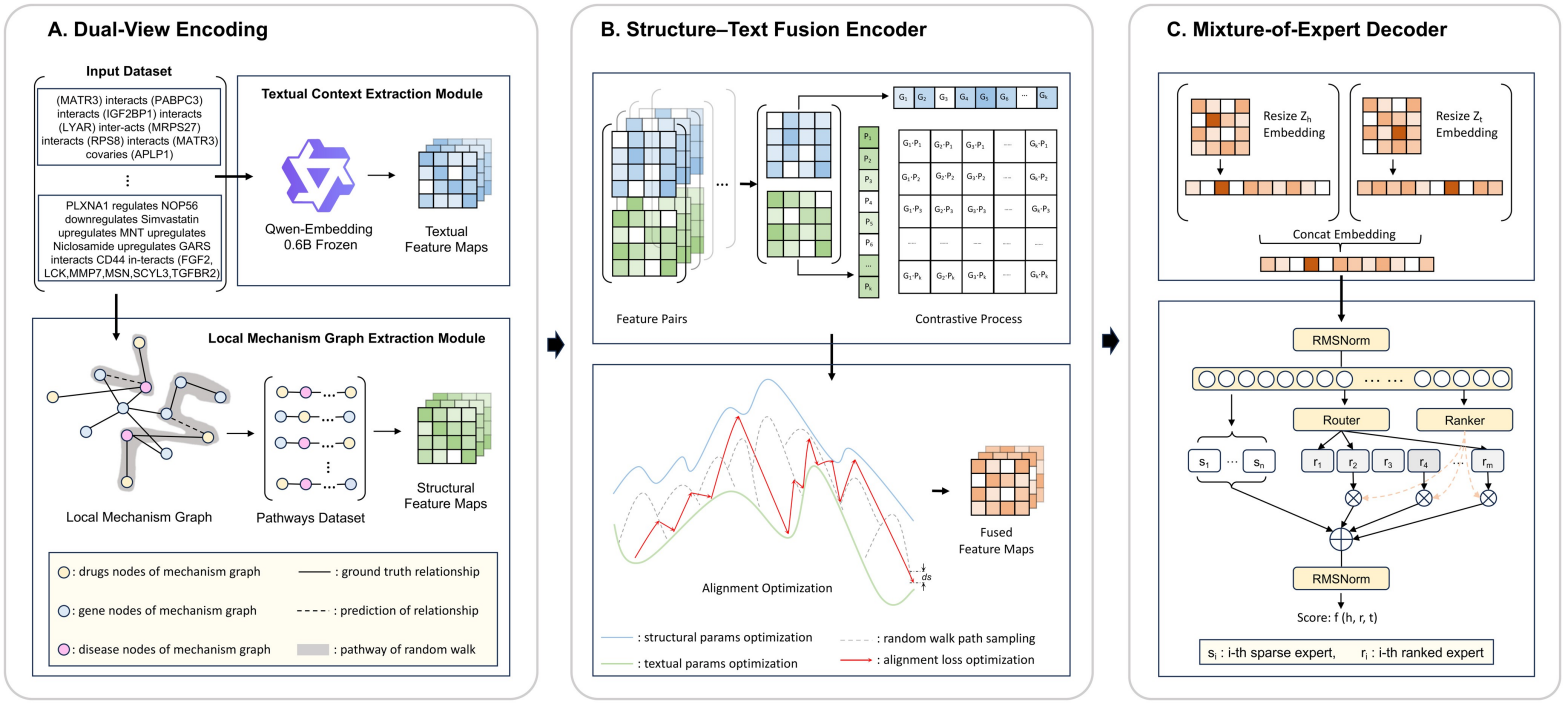
The KLaR architecture. (a) Dual-view encoding: for an entity *v*, KLaR extracts a local subgraph and generates textualized mechanism paths. (b) Gated fusion: structural embeddings ($s_{v}$) and textual embeddings ($t_{v}$) are aligned via contrastive learning and fused into $z_{v}$. (c) MoE inference: a sparse router dispatches the fused representation to relation-specialized experts to compute the final triple score.

The inference workflow (Fig. 1) processes a query entity *v* through two parallel views: (i) **Structural View**, where a relational GNN encodes the *k*-hop local subgraph $G_{v}$; and (ii) **Textual View**, where mechanism-consistent paths are linearized and embedded. These views are projected into a shared latent space via a gated fusion mechanism. Finally, to capture the heterogeneity of biomedical interactions (e.g. distinct patterns in metabolic pathways versus gene regulation), we use a sparse MoE decoder to predict link probabilities.

### 3.2 View I: mechanism-consistent textual encoding

To capture semantic context without the overhead of generative inference, we use a frozen pretrained textual encoder $\Phi :T\to R^{d_{\text{emb}}}$, where T denotes the token space and $d_{\text{emb}}$ is the embedding dimension.

In this work, we instantiate Φ with **Qwen3-Embedding** as the default frozen text encoder. The framework is compatible with other frozen sentence-embedding models, while the empirical sensitivity to encoder choice is evaluated separately in the experiments.

**Neighborhood textualization.** Instead of utilizing external unstructured text, which may introduce uncontrolled or weakly grounded semantic signals, we generate a textual corpus $C_{v}$ strictly derived from graph topology. Specifically, we sample a set of bounded-length random walks starting from *v*: $P_{v}=\{p_{1},\ldots ,p_{M}\}$. Each path $p=(v_{0}\overset{r_{1}}{\to }v_{1}\cdots \overset{r_{T}}{\to }v_{T})$ is linearized using relation-specific templates $T_{r}$:


$$\text{seq}(p)=\text{Concat}(v_{0},T_{r_{1}},v_{1},\ldots ,T_{r_{T}},v_{T}).$$

This design ensures that the textual input remains *mechanism-consistent* with the underlying biological interactions. The number of random walks *M* and the maximum path length *T* are treated as hyperparameters and are specified in Appendix 1.1, available as supplementary data at *Bioinformatics* online.

**Semantic aggregation.** Let $X_{v}$ denote the tokenized sequence of $C_{v}$. The textual representation $t_{v}\in R^{d_{t}}$ is obtained via mean pooling over the frozen encoder outputs:


$$t_{v}=\frac{1}{\left|X_{v}\right|}\sum_{x\in X_{v}}\Phi (x).$$

Gradients are not backpropagated through Φ, ensuring training efficiency and isolating semantic encoding from task-specific optimization.

### 3.3 View II: local mechanism graph encoding

To preserve local relational structure while maintaining computational efficiency, we extract the *k*-hop enclosing subgraph $G_{v}=(N(v),E_{v})$ for each target entity *v*. We use a Relational Graph Neural Network (RGNN) to encode this structure.

Let $h_{u}^{\left(\ell \right)}$ denote the embedding of node *u* at layer ℓ. The update rule aggregates messages from relation-specific neighborhoods:


$$h_{u}^{\left(\ell +1\right)}=\sigma \left(W_{0}^{\left(\ell \right)}h_{u}^{\left(\ell \right)}+\sum_{r\in R}\sum_{w\in N_{r}(u)}\alpha_{u,r,w}W_{r}^{\left(\ell \right)}h_{w}^{\left(\ell \right)}\right),$$

where $N_{r}(u)$ denotes neighbors connected via relation *r*, $W_{r}^{\left(\ell \right)}$ are relation-specific transformation matrices, and $\alpha_{u,r,w}$ is an attention coefficient capturing the importance of interaction (u,r,w) within the local biological context. The final structural embedding is $s_{v}=h_{v}^{\left(L\right)}\in R^{d_{s}}$. Specifically, attention scores are computed using a relation-aware additive attention function followed by softmax normalization over neighbors sharing the same relation type.

#### 3.3.1 Attention coefficient definition

The attention coefficient $\alpha_{u,r,w}$ is computed via a relation-aware additive attention mechanism:


$$\alpha_{u,r,w}={\text{softmax}}_{w\in N_{r}(u)}\left(\text{LeakyReLU}\left(a_{r}^{⊤}[W_{r}^{\left(\ell \right)}h_{u}^{\left(\ell \right)}||W_{r}^{\left(\ell \right)}h_{w}^{\left(\ell \right)}]\right)\right),$$

where $a_{r}\in R^{2d}$ is a relation-specific attention vector, ‖ denotes concatenation, and softmax is applied over all neighbors *w* sharing the same relation type *r*. This ensures that attention weights are normalized within each relation type, preserving the semantic specificity of different biomedical relations.

### 3.4 Structure–text fusion and alignment

Biomedical entities often exhibit a modality gap between structural interactions encoded in KGs and their semantic descriptions. From a biological perspective, effective fusion requires that both modalities refer to the same underlying local mechanism. We address this through a learnable gated fusion and explicit alignment objective.

**Gated integration.** Both structural and textual representations are projected into a unified dimension *d* via MLPs $\phi_{s}$ and $\phi_{t}$. A gating vector $\gamma_{v}\in {\left(0,1\right)}^{d}$ dynamically balances information flow:


$$\begin{array}{c} \gamma_{v}=\sigma (W_{\gamma }[s_{v};t_{v}]+b_{\gamma }),\;b_{\gamma }\in R^{d}\;\text{is}\;a\;\text{learnable}\;\text{bias}.\\ z_{v}=\gamma_{v}⊙\phi_{s}(s_{v})+(1-\gamma_{v})⊙\phi_{t}(t_{v}). \end{array}$$

**Contrastive alignment.** To enforce semantic consistency between structural and textual views, we maximize mutual information between $s_{v}$ and $t_{v}$ using an InfoNCE-style objective:


$$L_{\text{align}}=-\frac{1}{\left|B\right|}\sum_{v\in B}\;\text{log}\;\frac{\;\text{exp}(\text{sim}(\phi_{s}(s_{v}),\phi_{t}(t_{v}))/\tau )}{\sum_{u\in B}\;\text{exp}\;(\text{sim}(\phi_{s}(s_{v}),\phi_{t}(t_{u}))/\tau )}.$$

where B denotes the mini-batch and τ is a temperature parameter. The expectation over B provides a mini-batch estimate of the full-data objective, following standard contrastive learning practice.

### 3.5 Mechanism-specialized mixture-of-experts decoder

We introduce the Mixture-of-Experts (MoE) not as a generic ensembling technique, but as a mechanism-specialized inductive bias for modeling heterogeneous biomedical relations. Biomedical interactions exhibit diverse structural and semantic patterns (e.g. metabolic, regulatory, and clinical associations), which are difficult to capture using a single shared scoring function.

We use (u,r,w) to denote local edges within the *k*-hop subgraph during message passing, and (h,r,t) for global triples in the link prediction task. We define a set of *N* experts ${\left\{E_{k}\right\}}_{k=1}^{N}$, where each expert implements a lightweight relation scoring function (e.g. DistMult-style bilinear scoring).

For a given triple (h,r,t), we first compute router logits


$$a(h,r)={\text{MLP}}_{g}([z_{h};r_{r}])\in R^{N},$$

where ${\text{MLP}}_{g}$ is a two-layer MLP with hidden dimension d/2 and ReLU activation, $z_{h}$ is the fused representation of the head entity, and $r_{r}\in R^{d_{r}}$ is the trainable embedding of relation *r*.

We then retain only the Top-*K* experts according to the router logits a(h,r) and renormalize their weights with a softmax over the selected experts:


$$\pi_{k}(h,r)=\frac{\;\text{exp}(a_{k}(h,r))}{\sum_{j\in \text{Top}-K(a(h,r))}\;\text{exp}\;(a_{j}(h,r))},\;$$

The final triple score is


$$f(h,r,t)=\sum_{k\in \text{Top}-K(a(h,r))}\pi_{k}(h,r)E_{k}(z_{h},z_{t},r_{r}).$$

Each expert $E_{k}$ implements a DistMult-style bilinear scoring function with its own relation embedding $r_{r}^{\left(k\right)}$:


$$E_{k}(z_{h},z_{t},r_{r})=\langle z_{h},r_{r}^{\left(k\right)}⊙z_{t}\rangle ,$$

where ⊙ denotes element-wise multiplication. Experts are relation-type agnostic but specialize through different learned relation embeddings and the routing mechanism.

To prevent expert collapse and encourage balanced expert usage, we use an auxiliary MoE regularization term


$$L_{\text{moe}}=\lambda_{\text{load}}L_{\text{load}}+\lambda_{\text{imp}}L_{\text{imp}},$$

where $L_{\text{load}}$ balances expert utilization across a mini-batch and $L_{\text{imp}}$ discourages concentration of routing mass on only a few experts.

### 3.6 Optimization objective

The model is trained end-to-end by minimizing a joint objective comprising the max-margin ranking loss $L_{\text{rank}}$, the alignment loss, and the expert regularization:


$$L=L_{\text{rank}}+\lambda_{1}L_{\text{align}}+\lambda_{2}L_{\text{moe}}.$$

where $L_{\text{align}}$ is the cross-modal alignment loss and $L_{\text{moe}}$ is the expert regularization term. For link prediction, we use a max-margin ranking loss:


$$L_{\text{rank}}=\sum_{(h,r,t)\in B}\sum_{(h^{\prime },r,t^{\prime })\in N}\max\left(0,\gamma +f(h^{\prime },r,t^{\prime })-f(h,r,t)\right).$$

All subgraph extraction and training procedures strictly exclude test edges. We optimize all encoder and decoder parameters while keeping the Qwen3-Embedding module frozen throughout training. Additional implementation details, including hyperparameter settings and the full training algorithm, are provided in Appendix 1.1, available as supplementary data at *Bioinformatics* online.

## 4 Experiments

### 4.1 Experimental setup

#### 4.1.1 Datasets

We evaluate KLaR on three heterogeneous biomedical knowledge graphs: PharmKG, HetioNet, and DTINet. Unless otherwise stated, each dataset is split into 80%/10%/10% for train/validation/test. We follow the standard *filtered* link prediction protocol for KG models, where ranking is performed over the full entity set while filtering other true triples from the candidate list. A summary of entity and relation counts, as well as the sizes of each split, is provided in Table 8, available as supplementary data at *Bioinformatics* online. More fine-grained statistics of node and edge types (e.g. relation-type distributions in HetioNet, PharmKG, and DTINet) are also reported in the Supplementary Materials, available as supplementary data at *Bioinformatics* online.

#### 4.1.2 Negative sampling

During training, for each positive triple (h,r,t), we generate corrupted negatives by replacing either the head or the tail with equal probability. Unless otherwise stated, we sample 128 negative triples per positive example. We note that different baselines may adopt different negative sampling budgets in their original implementations; to improve reproducibility and reduce confounding factors, we report (i) results under each baseline’s official/default settings, and (ii) additional training-setting details and sensitivity analyses in the Supplementary Materials, available as supplementary data at *Bioinformatics* online.

#### 4.1.3 Evaluation metrics

We report Mean Reciprocal Rank (MRR), Hits@1, Hits@3, and Hits@10 under the filtered setting. All results are averaged over three random seeds; standard deviations are provided in the Supplementary Materials, available as supplementary data at *Bioinformatics* online to facilitate statistical comparison.

#### 4.1.4 Text embedding instantiation (frozen encoder)

For all KLaR experiments, we use **Qwen3-Embedding-0.6B** as a frozen sentence-embedding model. The encoder is used strictly as a feature extractor: we do not fine-tune its parameters, perform autoregressive generation, or use task-specific prompting for inference-time reasoning. While embeddings are produced through the encoder’s internal transformer computation, all task-specific relational reasoning in KLaR is performed by the lightweight fusion encoder and the MoE decoder. Consequently, the trainable component of KLaR is orders of magnitude smaller than full autoregressive LLM baselines (e.g. Qwen3-8B and LLaMA2-7B).

#### 4.1.5 LLM Baseline protocol (candidate-restricted ranking)

To ensure reproducibility, we evaluate text-only LLM baselines(BioWordVec, Qwen3-8B, LLaMA2-7B, DeepSeek) using a standardized JSON-based prompt (Appendix 1.3, Listing 1, available as supplementary data at *Bioinformatics* online). Each link prediction query is formatted as a multiple-choice question containing the head entity, relation type, and a candidate tail set of size $N_{\text{cand}}$. Since ranking over the full entity set is not computationally feasible for prompted LLM inference, LLM baselines are evaluated under a *candidate-restricted* protocol with $N_{\text{cand}}=100$ (including the ground-truth entity and sampled negatives), following a consistent corruption strategy across datasets. All LLMs operate in strict zero-shot mode without external tools, fine-tuning, or in-context examples, and we explicitly disable chain-of-thought reasoning (Appendix 1.3, Listing 2, available as supplementary data at *Bioinformatics* online). The model’s answer probabilities or logits are used for ranking candidates. We include these baselines as reference points for text-only reasoning under a controlled setting; they are not strictly equivalent to full filtered ranking over all entities.

#### 4.1.6 Reproducibility and missing baseline entries

Some baselines are not applicable to all KGs or may be infeasible under certain settings due to memory/time constraints. For transparency, we provide in the Supplementary Materials, available as supplementary data at *Bioinformatics* online a detailed feasibility table documenting per-baseline applicability, hyperparameters, and failure modes for missing entries in Table 1 (e.g. ProjE, HittER, DTD-GNN).

**Table 1:** Benchmark link prediction results on PharmKG, HetioNet, and DTINet.^a^

Mean reciprocal rank and hit rate (higher is better ↑)												
	PharmKG tasks				HetioNet tasks				DTINet tasks			
Models	MRR	*N* = 1	*N* = 3	*N* = 10	MRR	*N* = 1	*N* = 3	*N* = 10	MRR	*N* = 1	*N* = 3	*N* = 10
*(I) Filtered ranking over the full entity set (directly comparable KG-based results)*												
TransE	0.091	0.034	0.092	0.198	0.027	0.002	0.022	0.070	0.102	0.035	0.095	0.218
TransR	0.075	0.030	0.071	0.155	0.040	0.013	0.036	0.088	0.091	0.041	0.103	0.204
RotatE	0.071	0.023	0.057	0.161	0.023	0.002	0.012	0.054	0.010	0.001	0.004	0.014
RESCAL	0.064	0.023	0.057	0.122	0.032	0.017	0.031	0.059	0.078	0.031	0.082	0.173
ComplEx	0.107	0.046	0.110	0.225	0.070	0.029	0.069	0.148	0.138	0.063	0.149	0.326
ProjE	–	–	–	0.249	–	–	–	0.261	–	–	–	0.074
DistMult	0.071	0.023	0.062	0.157	0.029	0.006	0.022	0.067	0.026	0.006	0.017	0.051
Analogy	0.070	0.022	0.060	0.159	0.024	0.004	0.017	0.058	0.013	0.003	0.008	0.025
ConvE	0.086	0.038	0.087	0.169	0.075	0.032	0.071	0.155	0.127	0.047	0.141	0.241
ConvKB	0.106	0.052	0.107	0.209	0.094	0.045	0.090	0.186	0.157	0.061	0.163	0.371
RGCN	0.067	0.027	0.062	0.139	0.030	0.011	0.021	0.052	0.074	0.029	0.068	0.142
HittER	–	0.025	0.065	0.166	0.046	0.018	0.039	0.095	–	0.006	0.017	0.049
STaR	0.068	0.031	0.065	0.135	0.016	0.004	0.014	0.030	0.129	0.071	0.133	0.242
FuseLinker	0.169	0.075	0.177	0.372	0.133	0.057	0.137	0.286	0.232	0.090	0.246	0.605
KG-LLM	0.166	0.072	0.174	0.379	0.131	0.056	0.135	0.289	0.233	0.088	0.243	0.600
BioBLP	0.145	0.061	0.150	0.330	0.121	0.051	0.120	0.268	0.210	0.077	0.215	0.538
DTD-GNN	–	–	–	–	–	–	–	–	0.236	0.087	0.241	0.598
Qwen3-0.6B (fine-tuned)	0.164	0.067	0.155	0.339	0.092	0.047	0.121	0.194	0.212	0.062	0.221	0.421
KLaR	**0.182**	**0.089**	**0.194**	**0.396**	**0.146**	**0.069**	**0.151**	**0.306**	**0.254**	**0.103**	**0.261**	**0.621**
*(II) Candidate-restricted ranking (text-only reference results; not directly comparable)*												
BioWordVec	0.121	0.057	0.112	0.250	0.080	0.019	0.056	0.220	0.188	0.076	0.186	0.475
Qwen3-8B	0.159	0.069	0.170	0.383	0.125	0.054	0.123	0.277	0.214	0.080	0.219	0.546
LLaMA2-7B	0.158	0.068	0.169	0.350	0.124	0.053	0.122	0.274	0.213	0.079	0.218	0.543
DeepSeek	**0.162**	**0.068**	**0.180**	**0.360**	**0.127**	**0.055**	**0.125**	**0.292**	**0.218**	**0.081**	**0.222**	**0.607**

a **(I) Filtered ranking over the full entity set:** Structure-based and KG–language hybrid models (including KLaR) are evaluated using the standard filtered protocol and are directly comparable. **(II) Candidate-restricted ranking:** Text-only language model baselines are evaluated under a candidate-restricted protocol due to the infeasibility of full-entity ranking with prompted generation, and are reported as reference results only. Missing entries indicate infeasibility or inapplicability; details are provided in the Supplementary Materials, available as supplementary data at *Bioinformatics* online. Bold values indicate the best performance, and underlined values indicate the second-best performance.

Bold values indicate the best performance, and underlined values indicate the second-best performance.

### 4.2 Baselines

We compare KLaR with four categories of representative methods:

- **Structural KGE models:** TransE (Bordes *et al*. 2013), DistMult (Yang *et al*. 2015), ComplEx (Trouillon and Bouchard 2017), RESCAL (Nickel *et al*. 2011), RotatE (Sun *et al*. 2019), Analogy (Liu *et al*. 2017).
- **Neural KGE/GNN-based models:** ConvE (Dettmers *et al*. 2018), ConvKB (Nguyen *et al*. 2018), R-GCN (Schlichtkrull *et al*. 2018), HittER (Chen *et al*. 2021), STaR (Li and Yang 2022).
- **Large-scale language models (text-only):** BioWordVec (Chiu *et al*. 2016) and autoregressive LLMs [Qwen3-8B (Yang *et al*. 2025), LLaMA2-7B (Touvron *et al*. 2023), DeepSeek (Li *et al*. 2024a)].
- **KG–LM hybrid and multimodal biomedical models:** FuseLinker (Xiao *et al*. 2024), KG-LLM (Liu *et al*. 2024), BioBLP (Daza *et al*. 2023), DTD-GNN (Li *et al*. 2024b).

The first two groups focus on graph-derived structure; text-only baselines quantify the capability of pretrained language representations under candidate-restricted prompting; and KG–LM hybrids represent strong baselines that explicitly integrate structural and textual information. In contrast to full autoregressive LLM baselines (7B–8B parameters), KLaR uses the *embedding-only* variant **Qwen3-Embedding-0.6B** as a frozen feature extractor and performs task-specific relational reasoning through lightweight fusion and decoding modules. Accordingly, improvements in KLaR should be attributed to mechanism-consistent information fusion and relation-specialized decoding, rather than to scaling generative inference.

### 4.3 Overall performance

Table 1 summarizes link prediction performance on PharmKG, HetioNet, and DTINet. Across all three KGs, KLaR achieves the strongest overall performance among the evaluated KG-based methods, with consistent gains over competitive KG–LM hybrids such as FuseLinker (Xiao *et al.* 2024) and KG-LLM (Liu *et al.* 2024). On PharmKG, KLaR improves MRR from 0.169 (FuseLinker) and 0.162 (DeepSeek) to 0.182, and raises Hits@10 to 0.396. On HetioNet, KLaR improves MRR from 0.133 (FuseLinker) to 0.146. On DTINet, KLaR attains an MRR of 0.254, outperforming FuseLinker (0.232) and DTD-GNN (0.236), and achieves a Hits@10 of 0.621.

Because text-only baselines are evaluated under a candidate-restricted ranking protocol, we additionally assess sensitivity to the candidate budget around the default setting used in Table 1. As shown in Table 2, enlarging the candidate set from 50 to 200 predictably lowers MRR for all text-only models, while the relative ordering remains broadly stable. This supports our use of $N_{\text{cand}}=100$ as a representative reference setting, while also highlighting that these text-only results should not be interpreted as directly comparable to full filtered ranking over the entire entity set.

**Table 2:** Sensitivity of candidate-restricted text-only evaluation to the candidate set size $N_{\text{cand}}$ on **PharmKG**.^a^

Model	$N_{\text{cand}}=50$	$N_{\text{cand}}=100$	$N_{\text{cand}}=200$
BioWordVec	0.132	0.121	0.108
Qwen3-8B	0.176	0.159	0.144
LLaMA2-7B	0.174	0.158	0.142
DeepSeek	0.179	0.162	0.147

a Results are reported as MRR under the same prompt format and corruption strategy.

#### 4.3.1 Comparison with fine-tuned LM baseline

To further contextualize the benefits of our mechanism-consistent fusion approach, we fine-tuned a Qwen3-0.6B model directly on the link prediction task using the same training data. As shown in Table 1, the fine-tuned LM achieves competitive performance but still underperforms KLaR across all datasets. This suggests that: (i) domain-specific fine-tuning alone is insufficient to fully leverage structural information encoded in KGs; and (ii) explicit structure-text alignment at the subgraph level provides complementary benefits beyond what a fine-tuned text encoder can capture.

#### 4.3.2 Comparison with text-only LLMs

Autoregressive LLMs achieve strong performance under the candidate-restricted prompting protocol. These text-only results serve as controlled references under candidate-restricted evaluation and are not directly comparable to full filtered KG ranking. This suggests that mechanism-consistent information fusion between local structure and textualized neighborhood context can be an effective alternative to scaling generative inference for biomedical link prediction.

#### 4.3.3 Comparison with KG–LM hybrid models

**Figure 2:**
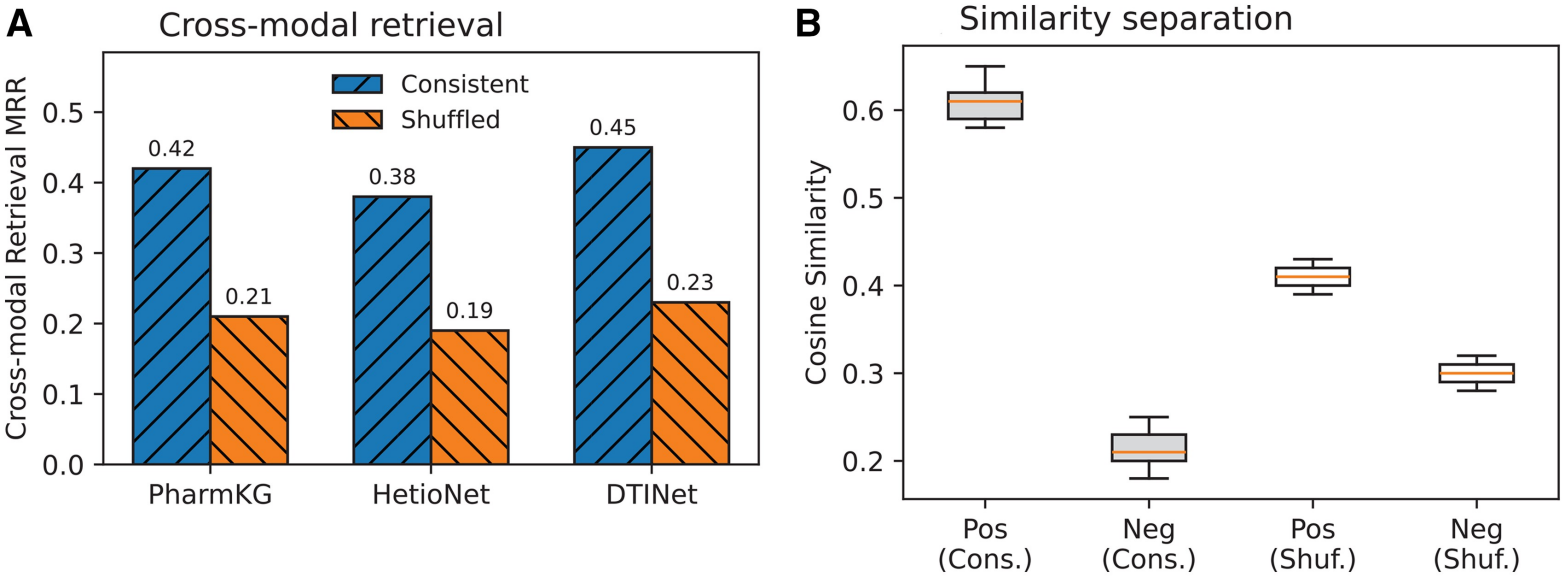
Mechanism-consistency analysis. (a) Cross-modal retrieval performance between structural and textual representations under mechanism-consistent textualization and a shuffled-text control. (b) Similarity distributions between matched and mismatched structure–text pairs.

Among all baselines, FuseLinker and KG-LLM are the closest competitors, reflecting the benefit of combining structural and textual representations on Fig. 2. DTD-GNN performs well on DTINet due to its tailored modeling of drug–target–disease graphs. KLaR consistently improves upon these strong hybrids, supporting the value of subgraph-level alignment and relation-specialized decoding beyond node-level description fusion.

#### 4.3.4 Comparison with structure-only KG models

Classical KGE methods (TransE, DistMult, ComplEx, etc.) and neural KGE/GNN-based models (ConvE, ConvKB, R-GCN, STaR) are weaker than KG–LM hybrid methods, particularly on heterogeneous biomedical graphs. This gap motivates principled integration of structural and semantic evidence for reliable biomedical link prediction.

We emphasize that increasing model size alone does not guarantee improved biomedical link prediction. Under the controlled prompting protocol, off-the-shelf 7B–8B LLMs do not consistently outperform KG–LM hybrids. In contrast, KLaR—built on a frozen embedding encoder and lightweight fusion/decoding modules—achieves strong performance while maintaining modest additional computational cost (Table 9, available as supplementary data at *Bioinformatics* online). For clarity, the reported model size corresponds to approximately 130M trainable parameters, excluding the frozen pretrained text encoder.

### 4.4 Mechanism-consistency analysis

While Table 1 demonstrates the effectiveness of KLaR on biomedical link prediction, we further examine whether the proposed mechanism-consistent textualization indeed induces more alignable structural and textual representations. To this end, we evaluate cross-modal retrieval and similarity separation between structural and textual embeddings, and compare against a shuffled-text control that breaks local mechanism-consistency.

### 4.5 Ablation studies

To better understand the contribution of each component in KLaR, we conduct ablation studies on all three datasets (Table 3).

**Table 3:** Ablation study of KLaR components on PharmKG, HetioNet, and DTINet.^a^

MRR and Hits@K (higher is better)												
Ablation	PharmKG				HetioNet				DTINet			
1 expert	0.155	0.062	0.157	0.364	0.113	0.045	0.108	0.254	0.203	0.071	0.199	0.526
2 experts	0.166	0.070	0.174	0.379	0.116	0.046	0.115	0.256	0.210	0.074	0.208	0.547
4 experts	0.163	0.069	0.170	0.370	0.119	0.049	0.118	0.259	0.224	0.082	0.229	0.580
8 experts	0.167	0.071	0.173	0.378	0.122	0.051	0.119	0.272	0.224	0.083	0.229	0.580
16 experts	0.171	0.075	0.181	0.385	0.126	0.053	0.124	0.280	0.227	0.085	0.233	0.585
32 experts	0.170	0.075	0.180	0.385	0.128	0.056	0.127	0.278	0.239	0.094	0.251	0.580
w/o $L_{\mathrm{load}}$	0.168	0.078	0.181	0.368	0.131	0.057	0.137	0.286	0.236	0.087	0.245	0.595
w/o $L_{\mathrm{importance}}$	0.169	0.074	0.180	0.370	0.135	0.058	0.140	0.288	0.239	0.089	0.248	0.603
w/o both	0.167	0.074	0.179	0.365	0.130	0.053	0.135	0.284	0.236	0.086	0.239	0.590
w/o MoE	0.168	0.073	0.178	0.378	0.121	0.052	0.124	0.275	0.230	0.086	0.237	0.588
w/o KGE	0.162	0.067	0.164	0.374	0.118	0.050	0.118	0.263	0.217	0.078	0.220	0.564
w/o TextE	0.169	0.075	0.181	0.382	0.132	0.056	0.135	0.290	0.236	0.090	0.246	0.603
w/o $L_{\text{align}}$	0.170	0.076	0.182	0.384	0.133	0.057	0.137	0.291	0.238	0.091	0.247	0.606
KLaR (64 Experts)	**0.182**	**0.089**	**0.194**	**0.396**	**0.146**	**0.069**	**0.151**	**0.306**	**0.254**	**0.103**	**0.261**	**0.621**

a We isolate the contribution of the MoE decoder (“w/o MoE”), the local graph encoding branch (“w/o KGE”), the textualized neighborhood context branch (“w/o TextE”), the structure–text alignment loss (“w/o $L_{\text{align}}$”), and expert regularization terms. Overall, combining mechanism-consistent fusion with relation-specialized decoding yields the strongest performance across datasets. Bold values indicate the best performance.

Bold values indicate the best performance.

#### 4.5.1 Effect of the MoE

Removing the MoE decoder (“w/o MoE”) and replacing it with a single shared scoring network reduces performance across datasets (e.g. MRR decreases from 0.182 to 0.168 on PharmKG and from 0.254 to 0.230 on DTINet), indicating that expert specialization is beneficial for modeling heterogeneous biomedical relation patterns.

#### 4.5.2 Effect of structural and textual branches

Removing the structural branch (“w/o KGE”) yields a clear performance drop (e.g. 0.182 → 0.162 MRR on PharmKG), highlighting that graph structure remains a primary source of evidence. Removing the textual branch (“w/o TextE”) also reduces performance (e.g. 0.182 → 0.169 on PharmKG), suggesting that textualized neighborhood context provides complementary information beyond the graph.

#### 4.5.3 Effect of structure–text fusion and alignment

Disabling the alignment loss (“w/o $L_{\text{align}}$”) consistently weakens results compared with the full model, indicating that explicit structure–text alignment stabilizes fusion and discourages over-reliance on any single modality.

#### 4.5.4 Effect of expert count and regularization

Increasing the number of experts generally improves performance, especially when moving from a single expert to multi-expert routing, after which gains gradually saturate. Removing expert regularization (load-balancing and importance losses) harms performance and leads to less stable training, consistent with its role in preventing expert collapse.

### 4.6 Biological case studies

**Figure 3:**
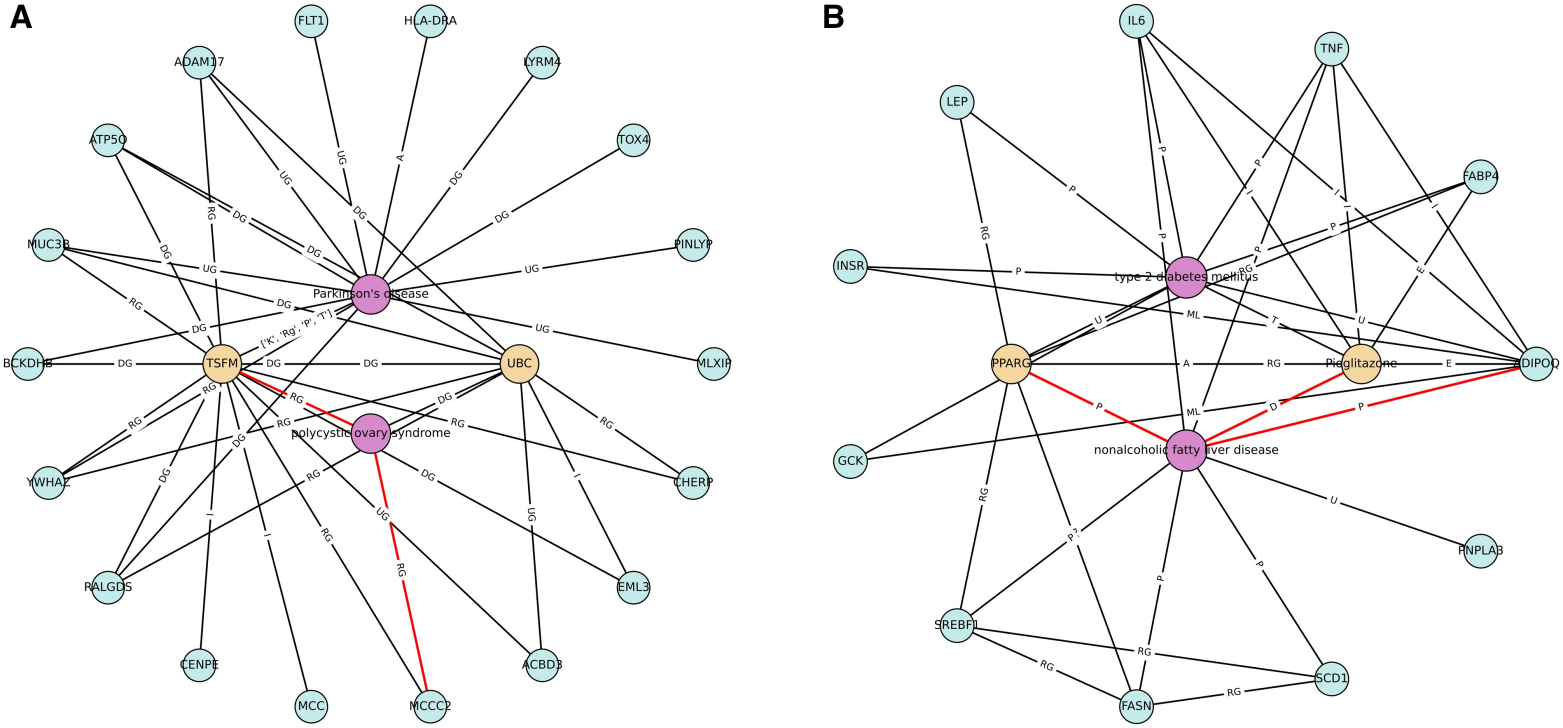
(a) **Case Study I**: KLaR predicts two missing PCOS–Gene associations (PCOS–*TSFM*, PCOS–*MCCC2*; red dashed edges), suggesting that PCOS engages a mitochondrial–regulatory module linked to Parkinson’s disease in the KG. (b) **Case Study II**: KLaR recovers missing NAFLD associations with *PPARG*, *ADIPOQ*, and *Pioglitazone* (red dashed edges), bridging a PPARG-centered metabolic module originally connected only to T2DM.

To evaluate whether KLaR can recover biologically meaningful relations *absent from the observed training graph*, we conduct two qualitative case studies (Fig. 3). In both modules, the dashed edges are missing from the original KGs yet ranked highly by KLaR. These examples illustrate how mechanism-consistent fusion can surface literature-supported associations that are under-represented in the structured graph.

#### 4.6.1 Case I: mitochondrial axis linking PCOS and Parkinson’s disease

Channel A centers on *PCOS*, *TSFM*, and *Parkinson’s disease*. Both PCOS and PD are increasingly recognized as disorders with pronounced mitochondrial abnormalities, including oxidative stress, respiratory-chain defects, and impaired mitophagy (Zhang *et al.* 2019, Finsterer 2023, Henrich *et al.* 2023). TSFM encodes a mitochondrial translation factor essential for oxidative phosphorylation (Emperador *et al.* 2017). Although the KG contains no direct PCOS–TSFM edge, KLaR ranks this association highly and highlights a coherent mitochondrial module linking PCOS-relevant genes to PD-related processes, consistent with emerging evidence on shared metabolic and mitochondrial disruptions. This case illustrates how KLaR can recover a plausible disease–mitochondria axis that is weakly represented in the structured graph.

#### 4.6.2 Case II: metabolic nuclear receptor axis linking NAFLD and type 2 diabetes

Channel B examines *NAFLD*, *T2DM*, and regulators *PPARG* and *ADIPOQ*. PPARγ governs adipocyte differentiation and insulin sensitivity, while adiponectin protects against hepatic steatosis and metabolic dysfunction (Cataldi *et al.* 2021, Dixon *et al.* 2021). PPARγ agonists such as pioglitazone improve NASH/NAFLD in clinical trials (Belfort *et al.* 2006). Although several NAFLD–PPARG/ADIPOQ edges are absent or sparse in the KG, KLaR ranks NAFLD–PPARG and NAFLD–ADIPOQ highly and connects them to a coherent metabolic axis consistent with established mechanisms, suggesting clinically relevant pathways involving druggable nuclear receptors.

Beyond these two modules, we provide additional randomly sampled examples in Tables 1–3, available as supplementary data at *Bioinformatics* online. Overall, the case studies indicate that KLaR not only improves link-prediction metrics but also surfaces biologically plausible relations that are missing or under-represented in the observed KGs.

## 5 Discussion and limitations

This work emphasizes principled information fusion rather than model scaling, and several design choices merit discussion. First, the sparse Mixture-of-Experts (MoE) decoder functions as a mechanism-specialized inductive bias rather than a standalone performance driver. Ablation results show that removing the MoE degrades performance, especially for heterogeneous relations, but does not collapse the model. This suggests that KLaR’s main gains arise from subgraph-level alignment of structural and textual evidence, with the MoE enabling flexible capacity allocation across interaction types.

Second, we use a frozen sentence-embedding model (Qwen3-Embedding-0.6B) to decouple semantic representation from task-specific reasoning and avoid domain-specific fine-tuning. Although alternative or domain-specialized encoders may affect absolute performance, the proposed framework is agnostic to the choice of text encoder. Exploring encoder variants and domain adaptation remains future work.

Finally, text-only LLM baselines are evaluated under a candidate-restricted protocol due to the infeasibility of full-entity ranking with generative inference. These results are therefore not directly comparable to filtered KG-based evaluations and are included only to contextualize text-only reasoning. KLaR targets standard KG link prediction scenarios requiring efficient ranking over large entity sets.

Overall, KLaR shows that mechanism-consistent fusion of local graph structure and textualized neighborhood context enables robust biomedical link prediction without reliance on large-scale generative inference. This approach complements advances in large language models by offering a controllable and efficient alternative for structured biomedical reasoning and hypothesis generation.

## Author contributions

Yinghui Jiang (Conceptualization, Methodology, Software, Validation, Formal analysis, Investigation, Resources, Data curation, Writing—original draft, Writing—review & editing, Visualization), Zixian Li (Validation, Formal analysis, Investigation, Data curation, Writing—original draft), Yanchao Xu (Validation, Formal analysis, Investigation, Data curation, Writing—original draft), Haotong Sun (Validation, Formal analysis, Investigation, Data curation, Writing—original draft), Bocheng Xu (Validation, Formal analysis, Investigation, Data curation, Writing—original draft), and Xiangrong Liu (Supervision, Project administration, Funding acquisition, Writing—review & editing)

## Supplementary Material

btag320_Supplementary_Data
