# Supplementary material for: KLaR: fusing knowledge graphs and language models for biomedical target discovery
Source: Bioinformatics. 2026 Jul 7;42(Suppl 1):btag320. doi: 10.1093/bioinformatics/btag320 (PMC13340231; doi:10.1093/bioinformatics/btag320)
Supplement: btag320_Supplementary_Data [file btag320_supplementary_data.pdf]

Supplementary Materials

Implementation Details

The experiments were conducted using a combination of high-performance computational resources to ensure the efficiency and scalability of the KLaR model. The specific configurations used are listed below:

- **Hardware Configuration:**
  - GPUs: NVIDIA Tesla A100 GPUs with 40 GB of memory each.
  - CPU: Intel Xeon Gold 6230 @ 2.10GHz.
  - RAM: 256 GB DDR4.
- **Software Libraries:**
  - PyTorch version 2.0.0.
  - PyTorch Lightning version 2.1.2.
  - Numpy version 1.24.1.
  - Scikit-learn version 0.24.1 for preprocessing and evaluation metrics.
  - Tqdm version 4.66.1 is used for estimating the remaining time.
- **Optimization and Regularization Strategies**:
  - We optimize KLaR using ADAM (Kingma and Ba, 2017) with weight decay (L2) , with a learning rate of $1e-4$. Adam is a widely used optimization method based on first and second order of moment.

Textualisation Embedding: We use Qwen3-Embedding-0.6B as a frozen sentence-embedding model. We apply the official tokenizer with a maximum length of $T_{\max}$ tokens, and use mean pooling over token embeddings to obtain t_v. No parameters of Qwen3-Embedding are updated during training.

Reproducibility: To ensure the reproducibility of our experiments, detailed information about the experimental setup is provided, including version numbers of tools listed in the requirement file, and detailed hyperparameter settings. The data split for training, validation, and testing datasets for different knowledge graphs can be found in Table S8.

Table S1 Detailed overview of cases within the DTI dataset, each with specific input and output labels. This table presents the results of KLaR on three randomly selected cases in the DTI task. The first column indicates the name of each case, the second column shows the input sequences fed into the model, and the third column displays the model's predicted outcomes.

Case	Label Input	Label Output
Case 1	SLC6A2 ASSOCIATED WITH seizures ASSOCIATED WITH ICAM1 COMPILED INTERACTS WITH Cocaine INTERACTS WITH Atropine INTERACTS WITH Dolasetron INTERACTS WITH Cevimeline INTERACTS WITH	Adefovir dipivoxil, Allopurinol, Streptomycin, Ifosfamide
Case 2	Phenoxymethylpenicillin INTERACTS WITH Levonorgestrel INTERACTS WITH Tipranavir INTERACTS WITH Pitavastatin INTERACTS WITH Oxybutynin INTERACTS WITH Theophylline INTERACTS WITH Esmolol INTERACTS WITH	Amyl Nitrite, Codeine, Dipyridamole, Frovatriptan
Case 3	SST ASSOCIATED WITH INTEGRATED optic nerve diseases ASSOCIATED WITH INTEGRATED SSTR2 COMPILED ACTS ON Protriptyline INTERACTS WITH Pancuronium INTERACTS WITH Prednisolone INTERACTS WITH Dofetilide INTERACTS WITH	Buprenorphine, Dopamine, Formoterol, Toremifene

Table S2 Detailed overview of cases within the HetioNet dataset, each with specific input and output labels. This table presents the results of KLaR on five randomly selected cases in the HetioNet task. The first column indicates the name of each case, the second column shows the input sequences fed into the model, and the third column displays the model's predicted outcomes.

Case	Label Input	Label Output
Case 1	MATR3 interacts PABPC3 interacts IGF2BP1 interacts LYAR interacts MRPS27 interacts RPS8 interacts MATR3 covaries (label)	APLP1
Case 2	PLXNA1 regulates NOP56 downregulates Simvastatin upregulates MNT upregulates Niclosamide upregulates GARS interacts CD44 interacts (label)	FGF2, LCK, MMP7, MSN, SCYL3, TGFBR2
Case 3	Lomitapide binds MTTP interacts PDIA4 regulates ERAP2 associates Crohn's disease associates DNAJC17 regulates TOMM70A regulates	ATF1, DHX29, IFRD2, IKZF1
Case 4	Dabrafenib downregulates PLAUR interacts VTN interacts ITGB6 interacts MAPK1 interacts CDT1 interacts HDAC11 regulates	EML3, PPP2R5E, PRR7
Case 5	USP25 interacts ANXA1 interacts SSRP1 interacts HARS interacts RPL12 interacts RSL1D1 downregulates obesity upregulates	B3GNT5, CFH, CXCL8, GPR183

Table S3 Detailed overview of cases within the PharmKG 8K task, each with specific input and output labels. This table presents the results of KLaR on three randomly selected cases in the PharmKG 8K task. The first column indicates the name of each case, the second column shows the input sequences fed into the model, and the third column displays the model's predicted outcomes.

Case	Label Input	Label Output
Case 1	nsd1 Relationship between genes nr2f2 Role in pathogenesis or promotes progression urologic neoplasms. Biomarkers (diagnostic) or regulation linked to disease. fgfr4 Role in pathogenesis or promotes progression head and neck neoplasms. Treatment or therapy: carboplatin Inhibits bax Relationships involving regulation and pathways.	akr1b1, casp2, ctcf, nr3c1
Case 2	myh11 Relationships involving regulation and pathways. notch1 Relationship between genes. psen2 Relationship between genes. casp1 Relationships involving regulation and pathways. ccl2 Role in pathogenesis or promotes progression of infertility. Biomarkers (diagnostic) or regulation linked to disease. lifr Enhance response or activate stimulate.	csf2rb, il6st, ppp2ca
Case 3	spermine Metabolism pharmacokinetics. egf Relationship between genes. ube2i Interactions with different types of entities. carcinoma bronchogenic Treatment or therapy. idarubicin Side effect or adverse event. stroke Biomarkers (diagnostic) or regulation linked to disease. tnfsf4 Enhance response or activate stimulate.	bank1, stat4

Table S4 Raw node and edge statistics of the HetioNet knowledge graph before task-specific preprocessing. This table reports statistics of the original HetioNet knowledge graph prior to any task-specific filtering or data splitting. The first section enumerates the counts of all node types (e.g., Compound, Gene/Protein, Disease, Pathway, Anatomy), while the second section reports the counts of relation types (e.g., Anatomy–downregulates–Gene and Anatomy–expresses–Gene). The "Total" row summarizes the overall number of nodes and relations in the raw graph.

Type of nodes	Count	Type of types	Count
Compound	1552	Anatomy–downregulates–Gene	102240
Gene / Proteins	20945	Anatomy–expresses–Gene	526407
Pathway	1822	Anatomy–upregulates–Gene	97848
Pharmacologic class	345	Compound–binds–Gene	11571
Biological Process	11381	Compound–causes–Side Effect	138944
Cellular Component	1391	Compound–downregulates–Gene	21102
Molecular Function	2884	Compound–palliates–Disease	390
Side effect	5734	Compound–resembles–Compound	6486
Symptom	438	Compound–treats–Disease	755
Disease	137	Compound–upregulates–Gene	18756
Anatomy	402	Disease–associates–Gene	12623
		Disease–downregulates–Gene	7623
		Disease–localizes–Anatomy	3602
		Disease–presents–Symptom	3357
		Disease–resembles–Disease	543
		Disease–upregulates–Gene	7731
Total	47031	Total	2250197

Table S5 This table provides a granular breakdown of node and edge counts within the DTINet knowledge graph. It is structured into two primary sections: "Type of nodes" and "Type of types." Under "Type of nodes," you will find counts for various node types like Drug, Protein, and Disease. Furthermore, under "Type of types," counts for different relationship types including Chemical-Chemical and Chemical-Disease are provided. The "Total" row aggregates the overall counts for nodes and types in the DTINet knowledge graph.

Type of nodes	Count	Type of types	Count
Drug	707	Drug-interacts-with-Drug	229938
Protein	1485	Drug-compiled-acts-on-Protein	22117
Disease	5316	Drug-compiled-interacts-with-Protein	11051
		Drug-associated with-Protein	1722
		Protein-compiled acts on-Protein	13558
		Protein-curated interacts with-Protein	6827
		Protein-associated with integrated-Disease	540459
		Protein-associated with-Disease	113419
		Protein-detected in pathology sample-Disease	21495
		Disease-has parent-Disease	2546
		Drug-act on-Disease	312421
Total	7804	Total	1275553

Table S6 This table provides a comprehensive account of the nodes and edges within the PharmKG knowledge graph. It encompasses a variety of node types, including Chemical, Gene, and Disease, as well as their diverse interrelationships such as Chemical-Chemical, Chemical-Disease, and more. The table serves as a foundational resource for understanding the intricate structure and complexity of the PharmKG knowledge graph, a crucial aspect of our research.

Type of nodes	Count	Type of types	Count
Chemical	1497	Chemical-Chemical	32100
Gene	4759	Chemical-Disease	68791
Disease	1347	Chemical-Gene	24297
		Disease-Chemical	451
		Disease-Disease	1599
		Disease-Gene	49199
		Gene-Chemical	7073
		Gene-Disease	68417
		Gene-Gene	249031
Total	7603	Total	500958

Table S7 This table summarizes relationship themes in the PharmKG knowledge graph by entity-type pair. The "Symbol" column denotes distinct themes, and the "Entity-type pairs" column specifies the associated pairs (e.g., Chemical–Gene).

Entity-type pair	Symbol	Relationship themes
Chemical-Gene	A	Agonism, activation, or antagonism, blocking
	B	Binding, ligand
	E	Affects expression/production
	N	Inhibits
Gene-Chemical	O	Transport, channels
	K	Metabolism, pharmacokinetics
	Z	Enzyme activity
Chemical-Disease	T	Treatment/therapy
	Sa	Side effect/adverse event
	C	Inhibits cell growth
	J	Role in pathogenesis
	Pr	Prevents, suppresses, or alleviates, reduces
Disease-Chemical	Mp	Biomarkers (progression)
Gene-Disease	P	Role in pathogenesis, or promotes progression
	Te	Possible therapeutic effect
	U	Mutations affect, or polymorphisms alter risk
	D	Possible therapeutic effect
Disease-Gene	ML	Biomarkers (diagnostic), or regulation linked to disease
	X	Possible therapeutic effect
Gene-Gene	Rg	Relationships involving regulation and pathways
	B	Binding, ligand
	Ra	Enhance response, or activate, stimulate
	Q	Production by cell population
	E	affects expression/production
	GG	Relationship between genes
Chemical-Chemical	CC	Relationship between chemicals
Disease-Disease	An	Ancestors of disease
	As	Associations between diseases

Table S8 Task-specific entity, relation, and split statistics used for link prediction after preprocessing. This table reports statistics of the entity and relation sets used in the link prediction task after task-specific preprocessing and train/validation/test splitting. Reported entity counts correspond to the effective entity set participating in link prediction, rather than the full set of nodes in the original knowledge graph. The third to fifth columns indicate the numbers of training, validation, and test triples, respectively.

Dataset	Ent	Rel	Train	Valid	Test
DTI	8,173	9	1,020,420	127,040	128,093
HetioNet	19,930	10	450,165	55,889	56,052
PharmKG_8K	7,260	25	348,832	43,116	43,532
Total	35,363	44	1,819,417	226,045	227,677

Table S9 Training and inference efficiency on PharmKG using a single NVIDIA A100 GPU. This table compares the training time, inference throughput, and GPU memory usage of different models on PharmKG. The reported parameter counts refer to trainable parameters only and exclude frozen components such as the pretrained text encoder. Textual embeddings are precomputed and cached prior to link prediction unless otherwise specified.

**Model**	**#Params (M)**	**Train time / epoch (min)**	**Total train time (h)**	**Inference (triples/s)**	**Peak GPU memory(GB)**
TransE	15	2.1	0.4	260,000	3.2
R-GCN	6	4.5	0.8	140,000	5.1
BioBLP	110	8.7	1.5	85,000	8.9
DTD-GNN	95	9.2	1.6	80,000	9.3
**KLaR (ours)**	**130**	**11.0**	**1.9**	**72,000**	**10.1**

Prompt of LLM Baseline

Listing 1. JSON-structured prompt used for evaluating text-only LLM baselines on biomedical KG link prediction. The system role defines a standardized query format for relation prediction tasks. Given a head entity, a relation, and a candidate list of tail entities, the LLM selects the single most plausible answer. This representation ensures reproducibility, model-agnostic evaluation, and consistent comparison across LLM variants.

```
{
  "role": "system",
  "content": {
    "description": "You are a biomedical knowledge assistant designed to answer structured link-prediction queries.",
    "task": {
      "type": "Multiple-choice relation prediction",
      "goal": "Given a head entity and a relation, select the most plausible tail entity from a candidate list."
    },
    "input_format": {
      "Head": "{HEAD_ENTITY}",
      "Relation": "{RELATION}",
      "Candidates": ["A. {CANDIDATE_1}", "B. {CANDIDATE_2}", "..."]
    },
    "output_requirement": "Respond with ONLY the letter of the selected option (e.g., 'A'). No explanation.",
    "output_format": {
      "Answer": "A / B / C / ..."
    }
  }
}
```

Listing 2. Strict zero-chain-of-thought (Zero-CoT) variant of the LLM prompt used for KG link prediction. The system enforces that the model outputs only the final answer letter without revealing intermediate reasoning steps, ensuring fair zero-shot evaluation across LLM baselines.

```
{
  "role": "system",
  "content": {
    "description": "You are prohibited from providing chain-of-thought reasoning. Output only the final answer letter
        .",
    "instruction": "If you attempt to produce intermediate steps, replace them with: 'Reasoning removed to comply
        with zero-shot evaluation.'",
    "output_format": {
      "Answer": "A / B / C / ..."
    }
  }
```

```
}
```

Listing 3. Example instantiation of our LLM evaluation prompt. This structured query is constructed from a KG test triple (Pioglitazone, treats, ?). The LLM selects the best candidate purely based on its internal biomedical knowledge.

```
{
  "Head": "Pioglitazone",
  "Relation": "treats",
  "Candidates": [
    "A. Type 2 diabetes mellitus",
    "B. Irritable bowel syndrome",
    "C. Osteoarthritis"
  ],
  "Expected_Output": { "Answer": "A" }
}
```

Table S10 Robustness of KLaR to the choice of frozen text encoder. All other components and training settings are kept unchanged.

Dataset	Frozen encoder	MRR	Hits@1	Hits@3	Hits@10
PharmKG	Qwen3-Embedding-0.6B	0.182	0.089	0.194	0.396
PharmKG	intfloat/e5-base-v2	0.176	0.083	0.187	0.384
HetioNet	Qwen3-Embedding-0.6B	0.146	0.069	0.151	0.306
HetioNet	intfloat/e5-base-v2	0.141	0.064	0.146	0.296
DTINet	Qwen3-Embedding-0.6B	0.254	0.103	0.261	0.621
DTINet	intfloat/e5-base-v2	0.247	0.097	0.254	0.607

Algorithm 1 Mini-batch Training of KLaR

Input: Knowledge graph $\mathcal{G} = (\mathcal{V}, \mathcal{E}, \mathcal{R})$, positive triples $\mathcal{E}^+$, number of epochs N_{epoch}, batch size B
Output: Trained parameters $\Theta = \{\theta_{\text{RGNN}}, \theta_{\text{fusion}}, \theta_{\text{MoE}}\}$
Initialise RGNN parameters θ_{RGNN}
Initialise fusion parameters $\theta_{\text{fusion}} = (\phi_s, \phi_t, W_\gamma, b_\gamma)$
Initialise MoE decoder parameters θ_{MoE} (experts, router, relation embeddings)
Load frozen textual encoder $\Phi = \text{Qwen3-Embedding}$
for $e = 1$ to N_{epoch} **do**
  Shuffle $\mathcal{E}^+$
  for mini-batch $\mathcal{B} \subset \mathcal{E}^+$ with $|\mathcal{B}| = B$ **do**
    Construct $\mathcal{B}'$ of positive and corrupted triples
    Let $\mathcal{V}_{\mathcal{B}'}$ be entities in $\mathcal{B}'$
    ▷ Structural view
    for each $v \in \mathcal{V}_{\mathcal{B}'}$ **do**
      Extract k-hop subgraph $G_v = (\mathcal{V}_v, \mathcal{E}_v)$
      Initialise node embeddings $\{\mathbf{h}_u^{(0)}\}_{u \in \mathcal{V}_v}$
      for $\ell = 0$ to $L - 1$ **do**
        for each $u \in \mathcal{V}_v$ **do**
          Update $\mathbf{h}_u^{(\ell+1)}$ by relation-aware message passing
          ▷ $\alpha_{u,r,w}$ computed via relation-specific attention, see Appendix S1.1
        end for
      end for
      $\mathbf{s}_v \leftarrow \mathbf{h}_v^{(L)}$
    end for
    ▷ Textual view
    for each $v \in \mathcal{V}_{\mathcal{B}'}$ **do**
      Sample bounded-length random walks on G_v
      Apply relation-specific templates T_r to obtain textual context C_v
      Tokenise C_v into $X_v = (x_1, \ldots, x_T)$
      $(\mathbf{e}_1, \ldots, \mathbf{e}_T) \leftarrow \Phi(x_1, \ldots, x_T)$ ▷ Φ frozen
      $\mathbf{t}_v \leftarrow \text{MeanPool}(\mathbf{e}_1, \ldots, \mathbf{e}_T)$
    end for
    ▷ Fusion and alignment
    for each $v \in \mathcal{V}_{\mathcal{B}'}$ **do**
      $\tilde{\mathbf{s}}_v \leftarrow \phi_s(\mathbf{s}_v)$, $\tilde{\mathbf{t}}_v \leftarrow \phi_t(\mathbf{t}_v)$
      $\gamma_v \leftarrow \sigma(W_\gamma[\mathbf{s}_v \| \mathbf{t}_v] + b_\gamma)$
      $\mathbf{z}_v \leftarrow \gamma_v \odot \tilde{\mathbf{s}}_v + (1 - \gamma_v) \odot \tilde{\mathbf{t}}_v$
    end for
    Compute InfoNCE alignment loss $\mathcal{L}_{\text{align}}$
    ▷ MoE decoding
    for each $(h, r, t') \in \mathcal{B}'$ **do**
      Retrieve $\mathbf{z}_h$, $\mathbf{z}_{t'}$, relation embedding $\mathbf{r}_r$
      Compute router logits $G(h, r) \leftarrow W_g[\mathbf{z}_h; \mathbf{r}_r]$
      Select Top-K experts with weights $G(h, r)_k$
      $f_\Theta(h, r, t') = \sum_{k \in \text{Top-}K} G(h, r)_k \, E_k(\mathbf{z}_h, \mathbf{z}_{t'}, \mathbf{r}_r)$
    end for
    Compute MoE regularisation loss $\mathcal{L}_{\text{moe}}$
    ▷ Loss and update
    Compute ranking loss $\mathcal{L}_{\text{rank}}$
    $\mathcal{L} \leftarrow \mathcal{L}_{\text{rank}} + \lambda_1 \mathcal{L}_{\text{align}} + \lambda_2 \mathcal{L}_{\text{moe}}$
    Update Θ using Adam with $\nabla_\Theta \mathcal{L}$
    ▷ Text encoder Φ is not updated
  end for
end for

Table S11 Performance on a redundancy-controlled test subset after filtering semantically redundant triplets.

Dataset	Model	MRR	Hits@1	Hits@3	Hits@10
PharmKG	FuseLinker	0.154	0.066	0.161	0.349
PharmKG	KG-LLM	0.150	0.063	0.158	0.344
PharmKG	KLaR	0.168	0.077	0.180	0.372
HetioNet	FuseLinker	0.121	0.049	0.124	0.268
HetioNet	KG-LLM	0.119	0.047	0.122	0.262
HetioNet	KLaR	0.134	0.060	0.139	0.287
DTINet	BioBLP	0.194	0.067	0.198	0.506
DTINet	FuseLinker	0.215	0.079	0.226	0.569
DTINet	KLaR	0.236	0.089	0.245	0.590

Table S12 Stress test with contradictory textual evidence. "Consistent" uses the original mechanism-consistent textual context; "Contradictory" replaces it with mismatched text while keeping graph structure fixed.

Dataset	Setting	MRR	Hits@1	Hits@3	Hits@10
PharmKG	KLaR (consistent)	0.182	0.089	0.194	0.396
PharmKG	KLaR (contradictory text)	0.171	0.078	0.181	0.377
PharmKG	w/o KGE (contradictory)	0.144	0.056	0.151	0.338
HetioNet	KLaR (consistent)	0.146	0.069	0.151	0.306
HetioNet	KLaR (contradictory text)	0.137	0.061	0.143	0.289
HetioNet	w/o KGE (contradictory)	0.110	0.041	0.113	0.244
DTINet	KLaR (consistent)	0.254	0.103	0.261	0.621
DTINet	KLaR (contradictory text)	0.242	0.093	0.249	0.598
DTINet	w/o KGE (contradictory)	0.206	0.069	0.209	0.529
